# The quest for a framework for sustainable and institutionalised priority-setting for health research in a low-resource setting: the case of Zambia

**DOI:** 10.1186/s12961-017-0268-7

**Published:** 2018-02-17

**Authors:** Lydia Kapiriri, Pascalina Chanda-Kapata

**Affiliations:** 1 Department of Health and Aging, McMaster University, 1280 Main Street West, Hamilton, Ontario Canada; 2 Department of Diseases Surveillance Control and Research, Ministry of Health, Lusaka, Zambia

**Keywords:** Priority-setting for health research, Approaches, Low-income countries, Zambia

## Abstract

**Background:**

Priority-setting for health research in low-income countries remains a major challenge. While there have been efforts to systematise and improve the processes, most of the initiatives have ended up being a one-off exercise and are yet to be institutionalised. This could, in part, be attributed to the limited capacity for the priority-setting institutions to identify and fund their own research priorities, since most of the priority-setting initiatives are driven by experts. This paper reports findings from a pilot project whose aim was to develop a systematic process to identify components of a locally desirable and feasible health research priority-setting approach and to contribute to capacity strengthening for the Zambia National Health Research Authority.

**Methods:**

Synthesis of the current literature on the approaches to health research prioritisations. The results of the synthesis were presented and discussed with a sample of Zambian researchers and decision-makers who are involved in health research priority-setting. The ultimate aim was for them to explore the different approaches available for guiding health research priority-setting and to identify an approach that would be relevant and feasible to implement and sustain within the Zambian context.

**Results:**

Based on the evidence that was presented, the participants were unable to identify one approach that met the criteria. They identified attributes from the different approaches that they thought would be most appropriate and proposed a process that they deemed feasible within the Zambian context.

**Conclusion:**

While it is easier to implement prioritisation based on one approach that the initiator might be interested in, researchers interested in capacity-building for health research priority-setting organisations should expose the low-income country participants to all approaches. Researchers ought to be aware that sometimes one shoe may not fit all, as in the case of Zambia, instead of choosing one approach, the stakeholders may select desirable attributes from the different approaches and piece together an approach that would be feasible and acceptable within their context. An approach that builds on the decision-makers’ understanding of their contexts and their input to its development would foster local ownership and has a greater potential for sustainability.

**Electronic supplementary material:**

The online version of this article (10.1186/s12961-017-0268-7) contains supplementary material, which is available to authorized users.

## Background

The limited resources available for global health research require that research priorities are set to identify the topics or issues that most urgently require study. Since setting priorities is ultimately about resource allocation, it often becomes a highly politicised process. This is more so in low-income countries (LICs), where the lack of resources to fund health research introduces non-state stakeholders whose research agendas may not necessarily be aligned with the national priorities. This makes it critical for LICs to strengthen capacity and increase funding for their national health research systems to enable them to set and fund national health priorities based on systematic processes using clear approaches with explicit criteria [1–5].

To facilitate priority-setting (PS) for health research, several frameworks have been developed. These include the Combined Approach Matrix (CAM) [6], the Essential National Health Research (ENHR) method [7], and the Child Health and Nutrition Research (CHNRI) approach [8, 9]. Further, two frameworks which have been used predominantly in high-income countries include Listening for Direction (L4D) [10] and the James Lind Alliance (JLA) method [11].

In addition to developing frameworks for PS in health research, there is an increasing interest in developing and strengthening the health research infrastructure by introducing national health research organisations/authorities and systems ((NHROs) in LICs; these should be responsible for, among other duties, setting the national health research priorities. This is because of the realisation that localised PS can contribute to ensuring that the research priorities are aligned with the local needs, increase the likelihood that the research findings will be used, facilitate dialogue between researchers, research funding organisations, policy-makers and implementers, and prevent duplicity of research projects and hence resource wastage. This also provides an opportunity for aligning PS to existing national planning systems and infrastructure [4, 5].

To date, several LICs have instituted NHROs [12]. However, not many of these have been sustainable, possibly due to lack of capacity and financial support [13]. Capacity-building for NHROs involves both the institutionalisation and the integration of the National Health Research Agenda in the national health policies, in addition to ensuring that resources are available for implementing the identified research priorities [14]. One way through which the NHROs’ capacities could be strengthened is by facilitating South–South collaboration, whereby LIC NHROs can harness, synthesise and share their experiences, identifying the limitations of the processes and devising improvement strategies. There are opportunities for countries currently developing their own NHRO to learn from the experiences of countries that have already established NHROs. For example, researchers from South Africa, a country with a long standing and as well as a well-functioning NHRO, could share their experiences with countries such as Zambia, which are in the process of developing their NHRO [15].

Zambia established the National Health Research Advisory Committee in 2005 to provide ad hoc support to the Zambian Ministry of Health on all matters relating to health research. In November 2008, the Committee began a participatory process of creating a new national institution designed to govern health research in the country [16]. In 2010, the Zambian government approved the National Health Research Policy; one of its recommendations was the formation of a national body to coordinate health research. By 2013, the government passed legislation providing a legal framework for the creation of the National Health Research Authority (NHRA), an entity designed to coordinate, fund and provide ethical oversight for health research, in addition to offering capacity strengthening to all stakeholders involved in research, advocating for the role of research in the health policy process and conducting PS exercises [17]. The acting co-ordinator of the temporary research authority identified the need for capacity strengthening within their institution; they thought it critical that there is robust support for this new entity to carry out its mandate. A critical step in ensuring the success of the NHRA is to ensure that the stakeholders involved have the necessary resources and the capacity to credibly set priorities for health research.

Capacity-building and availability of resources are critical to ensuring the sustainability and legitimacy of any priority-setting institution [18]. Capacity-building should involve members of an organisation that have the knowledge and ability to apply the available approaches/frameworks and evidence to their PS. However, while there are many resources devoted to facilitating health research PS (HRPS) in LICs, these resources are often not easily available or accessible to the people tasked with implementation. In Zambia, for example, where a systematic PS process was project based and led by consultants or international experts, resource constraints (including time) did not allow for sustained capacity strengthening for the NHROs [19]. In prioritisation processes that have not followed the COHRED process, only one PS approach is explained in detail and applied, for example, in the case of CHNRI applications [9]. This does not allow for the stakeholders involved to learn about other approaches. This lack of capacity-building makes it difficult for the NHRO to independently set health research priorities without the assistance of external experts. We argue that the tendency to depend on the external (and often expensive) experts to facilitate the process results in a vicious cycle of dependence, an inability to set research priorities if the resources are limited to hire the expert and a lack of sustainability of the activities of the NHROs.

There is a paucity of literature that addresses capacity-building for HRPS in LICs. While studies identify limited capacity as one of the main weaknesses of NHROs in LICs, existing capacity strengthening efforts have not involved empowering the NHROs with the necessary evidence enabling them to critically evaluate the available frameworks and assess what aspects of the approaches would be applicable, acceptable and feasible within their contexts. In this paper, we argue that the sustainability of NHROs in LICs will, in part, be facilitated by ensuring that the local technical staff in the NHRO in LICs have the necessary capacity to set health research priorities. Part of the capacity-building process should involve educating NHROs (and the relevant stakeholders) on all the available HRPS approaches, where they have been used and their strengths and limitations. This would enable NHROs to critically consider all the PS approaches for health research and to choose the most appropriate approach (or a hybrid of desired components from the different approaches).

Such an inclusive and comprehensive approach to capacity-building will contribute to ensuring ownership of the PS process, therefore facilitating a sustained application of the approach that emerges by stakeholders; such a method would also facilitate the institutionalisation of the chosen approach.

This paper reports findings from a pilot project in Zambia whose aim was to develop a systematic process for identifying components of a locally desirable and feasible HRPS approach and to contribute to capacity strengthening for the Zambia NHRA.

## Methods

This was a qualitative study involving literature review and systematic facilitated workshop discussions.

### Literature review

LK, a consultant and a research assistant participated in retrieving, reviewing and synthesising grey and published literature (at the global level) on the most commonly used HRPS approaches and their application from 1997 to 2014. The published literature was accessed using PubMed and Ovid databases (including Embase, Global Health, Medline, PsycINFO). The main search terms included different combinations of ‘priority setting’, ‘approaches’, ‘frameworks’ ‘health research’, ‘low income countries’ and ‘developing countries’.

### Step 2

A total of 1005 hits were derived from the different combinations of our search terms; these were initially scanned based on their titles to exclude papers that did not fit our criteria, e.g. those that did not address health research. Once we had identified all papers corresponding to our search strings and our date range, we applied the following inclusion and exclusion criteria in order to arrive at a shorter list of abstracts to review.

### Inclusion criteria

English language papers that appeared to describe a PS for health research framework^1^ or a case-study application of a PS for health research framework in any context (e.g. in a high-income country, middle-income country or LIC) or specific elements (e.g. stakeholder engagement, criteria generation, evaluation) stemming from either a PS for health research framework or a case study application were included in the study.

### Exclusion criteria

Papers that appeared to focus on PS for health interventions, describe a PS exercise that did not use an explicit PS framework for guidance or were in other languages were excluded.

### Step 3

A total of 108 papers met the inclusion criteria and were included in our literature review (a full reference list is included as Additional file 1). We then devised a ‘literature review framework’ that assigns the literature into one of three different categories, posing specific questions about the literature to help identify important trends, strengths and weaknesses. Table 1 below shows the three categories, the numbers of papers selected for full review (by category) and then a selection of the questions used to explore each literature category.

**Table 1:** Number of papers included in the literature by category

Category of paper	Description	Number of papers
1. PS Framework: concepts, theory, steps	These papers are largely theoretical or conceptual in nature; they describe, for instance, who developed a specific PS framework and why, the steps required in applying a framework, considerations of the stakeholders to involve, the use of criteria, and so on	23
2. PS Framework: application	These papers described case studies of health research PS exercises that applied the different frameworks; the focus here was on the reported experiences with the use of the frameworks, the documented strengths and limitations	59
3. PS Framework: specific elements	These are elements relevant to health research PS such as stakeholder engagement, criteria generation, use of evidence and evaluation (strengths and limitations) stemming from either a PS for health research framework or a case study application	26
Total		108

*PS* priority setting

This literature was supplemented with information from the websites of the organisation (such as COHRED, CHNRI, JLA) that developed the HRPS frameworks. PK and the research assistant in Zambia accessed the grey literature on HRPS within sub-Saharan Africa.

We summarised this information according to the above themes, as follows: What are the components of the framework? How was it developed and/or introduced? Where has the framework been used? What are the experiences with using the framework? What are the documented strengths and limitations of the framework?

### Validation of the synthesis

To formally validate our interpretation of the literature, the literature synthesis reports were sent to the different experts who developed and applied the frameworks. For the purposes of this study, we contacted experts from CAM (*n* = 1), CHNRI (*n* = 2), ENHR (*n* = 1), JLA (*n* = 2), and L4D (*n* = 1), specifically requesting them to assess whether our presentation of their framework was appropriate and to assess if we had missed any key literature related to their framework. We received responses from all experts with the exception of L4D. Their feedback and comments were used to edit the literature review report that was used in facilitating the capacity-building workshops.

### Capacity-building workshops

Two capacity-building workshops with different groups of stakeholders from Zambia were convened in July and November 2015. Both workshops involved researchers and policy-makers from the Zambia Ministry of Health. The second workshop also involved the board members of the Zambia NHRO and local researchers. The researcher participants are co-investigators on this research project. They identified policy-makers who were relevant to HRPS in Zambia.

#### Workshop I

The purposes of the workshop were to strengthen participants’ capacity for HRPS and to identify the desired PS attributes and processes that are best suited for implementation and sustainability within the Zambian context.

***The process*** *– capacity strengthening*: participants included three Ministry of Health officials from Zambia (who were also researchers) and four Canadian researchers. The approach was participatory, with shared responsibilities from both Zambian and Canadian researchers. LK led the first part of the meeting by asking participants about their experience with HRPS and their knowledge of HRPS frameworks. The Zambian participants reported and described a previous PS process in which they had participated. However, since they were only introduced to the CHNRI methodology, their understanding of the other HRPS frameworks was still limited. Hence, the first step in the capacity-strengthening process was to ensure that all workshop participants understood the commonly used HRPS frameworks, and the available evidence on their application.

Step 1:   LK presented descriptions of the five most common PS approaches in the literature – CAM, CHNRI, ENHR, JLA and L4D – and discussed the components and theoretical underpinnings of the HRPS approaches.
Step 2:   Based on Step 1, and using flip charts with the names of the different approaches, each participants was asked to independently indicate, on the respective flip charts, the perceived strengths and limitations for each framework (as in Table 1).
Step 3:   LK and PK presented and discussed the strengths and limitations of the approaches that were identified in the literature.
Step 4:   The Zambian participants identified the desired attributes and processes for HRPS in the Zambian context, based on the discussion in steps 2 and 3. Each attribute of each framework was discussed based on the following questions: would this [attribute or process] be desirable in the Zambian context? Would it be feasible? If not, would it require modification? The output of this process was a desired approach to HRPS and the key related attributes (summarised in Table 3). The responses to the questions were summarised and presented in the Results section.

The meeting proceedings were audio recorded with permission from all participants.

#### Workshop II

The second workshop was convened in Zambia and facilitated by the Zambian investigators who had participated in the first workshop. It involved six senior officers from the Ministry of Health in Zambia (two of whom were also researchers), and six university researchers who were identified by the Zambian co-investigator by virtue of their involvement in the Zambian NHRO. Most had also participated in the CHNRI PS exercise and therefore had prior experience with participatory decision-making and were also familiar with HRPS. The objective of this meeting was to validate the PS approach that was developed during the first workshop.

Prior to the workshop, all participants were sent invitation letters with an information package containing the workshop objectives and the proposed framework. They were requested to independently reflect on the different components of the proposed framework, and if they were indeed desirable within the Zambian context.

During the 1-day face-to-face meeting, after self-introductions (where participants also explained their knowledge of HRPS), the facilitators presented the objectives of the workshop. This was followed by a facilitated review of the proposed PS process for Zambia. Next, the participants were split into three groups and were requested to (1) review the proposed framework, step by step; (2) propose improvements or changes; and (3) present the main findings of their peer-review to all participants. The recommendations from each group were reviewed and consensus was sought on the final recommendations to be included in the proposed PS process for Zambia.

This study received ethics clearance from both Zambia and McMaster Research ethics boards. We obtained consent from all the group participants, and all findings were anonymised in the recording and reporting.

## Results

### Strengths of the five PS approaches

Table 2 summarises the strengths of the commonly used approaches from the literature and as perceived by the workshop participants. The most commonly identified strengths (in the literature and by the workshop participants) included the clarity and simplicity of the approach, involvement of a wide range of stakeholders, and explicit processes and criteria.

**Table 2:** Summary of the strengths of the five priority-setting frameworks

PS approach	Summary of the steps involved	Perceived strengths	
		From the literature	As identified by the workshop participants in addition to/support of those in the literature
ENHR	Step 1: How big is the health problem? Step 2: Why does the disease burden persist? Step 3: Is there sufficient knowledge about the problem to consider potential interventions? Step 4: How cost-effective will these interventions be? How soon can they be developed at a reasonable cost? Step 5: What are the current investments and available resources in this research area?	• Inclusiveness, involvement of a broad range of multidisciplinary, cross-sectoral stakeholders, e.g. experts, researchers, healthcare providers and representatives of the community • Transparent and systematic, involves analyses of health needs, and societal and professional expectations • Strong guiding principles, includes putting country priorities first, working for equity in development, and linking research to action for development • Provides a detailed listing of priority options	• The situation analysis provides opportunities to identify benchmarks for evaluation, is well aligned with already existing systems within the country • Provides chance to categorise the different service delivery opportunities • Creation of a consultative group or TWG based on the situation analysis
CHNRI	Step 1: PS framework managers or initiators identify and convene a group of experts or TWG Step 2: TWG members systematically create a long list of competing research options Step 3: Members of the TWG independently score the list of research options using specific criteria Step 4: PS framework managers and the TWG identify a Larger Reference Group to assess the importance of the criteria by attributing weights to the criteria Step 5: After applying the weights, the average scores for each research option are often, though not always, calculated to obtain the RPS Step 6: In some cases, the TWG then uses the derived RPS score to perform Program Budgeting Marginal Analysis with regards to research funding and the impact of the different options	• Reliability: the process is well documented and listed priorities are reproducible, challengeable, revisable • The process is systematic and reduces the impact of self-interest when deriving the initial list • Incorporates the consideration of values of a wider group of stakeholders and the public • It is transparent • Individual ranking in the technical working groups reduces any undue individual influence on the process and outcome • Methods can simultaneously evaluate different kinds of research, e.g. health systems research, intervention research	Participatory • TWGs bring experts together to discuss existing evidence, fosters a sense of ownership • Method recognises the extreme importance of multi-stakeholder engagement, makes the final priorities credible and acceptable to stakeholders • Face-to-face engagement of experts is very valuable, you would not get the same feeling virtually The process • Situation analysis ensures that current efforts/actors/evidence are part of the discussion • Standardised process using explicit criteria • Has the potential to offer a rapid assessment platform • Contextual applicability Priorities can be focal, i.e. research questions, or broad, i.e. research issues • Tried and tested in the local context and in other LICs • Uses most commonly used PS criteria • Aligned with the Ministry of Health policy-making process, and can be integrated in the system
JLA	Step 1 – Initiation: Establish a PSP of clinicians, patients and caregivers responsible for identifying, prioritising and publicising (the methods and results) identified priorities to the general public and research funders Step 2 – Gather uncertainties: The PSP then identifies and gathers a long list of the treatment uncertainties as perceived by patients, carers and clinicians, using an open or broad prompt such as “What questions about treating X would you like to be answered by research?” Prioritise the uncertainties: This involves two stages: Step 3 – Interim PS: This interim measure provides a short list of the uncertainties, and can be done by the steering committee or the PSP itself Step 4 – Final PS: This is often a face-to-face meeting or workshop, providing participants with an opportunity to express their views, hear those of others and broaden their thinking, often using the Nominal Group Technique in small groups or alternatively with larger group discussions, the aim is to develop an agreed-upon list of the top 10 priorities Step 5: Disseminate the final priorities to funders	• Integrating quantitative and qualitative methods (where applied) enables researchers to gather many validated uncertainties and to understand the rationale behind them • In 2009, the JLA underwent a formal review • Employs innovative and participatory approaches to involve patients in decision-making • Generates a list of priorities • Detailed description of how to engage the various stakeholders	Participatory • Engaging various stakeholders, e.g. the ‘public’ (specifically patients) • Proposes specific strategies for gathering input from the major stakeholders (e.g. electronically, face-to-face) The process • Streamlined and straightforward process • Clear idea of which stakeholders ought to participate • Explicitly define the use of the Nominal group technique or Delphi method to determine between competing priorities • Provides opportunities to revise and refine priorities
CAM	Step 1: Provide the best available information to participants who are populating the matrix; a comprehensive lack of information may indicate a research gap Step 2: Start by completing the public health dimension, e.g. magnitude of the problem, and work with the best available data (which may be national or global) before proceeding to the determinants row (for the different levels) Step 3: Discuss the entries; once all participants are in agreement, proceed to completing the third dimension Step 4: Review the entries in the two dimensions using the lens of the equity stratifier; participants should look at disaggregated data to assess if the problem is experienced differently by the stratified groups, and if so, identify the determinants; complete these cells once in agreement Step 5: Repeat this step for all the stratifiers Step 6: Distribute the final report to all relevant stakeholders; the 3DCAM should be applied in a PS process based on three equally important pillars: the PS process, the tools and the context	• Flexibility: can be applied in diverse contexts, for diverse issues, and by people of differing expertise • Practical and standardised way through which data can be presented and summarised, improving the transparency of the PS process • Organises, summarises and presents the best available information on one disease, risk factor, group or condition, and facilitates comparisons between the likely benefits of different types of intervention at different levels; this ensures that decisions are based on the best available evidence rather than participants’ views and knowledge • Draws attention to various domains where interventions are possible and desirable (from the household to global macroeconomic policies) • Explicit consideration of equity as a major dimension	Participatory workshops • This is already a strong feature of the health system in Zambia • TWGs and inclusion in the process is a part of the Zambian health system The process • Selection of priorities is a continuous and cyclical activity, this may facilitate institutionalisation • Ongoing structured tools for data collection to help inform the process of PS • Sampling the representativeness in terms of performance of health indicators at all levels (community, district, provincial, national) • Cost effectiveness and financial flows are considered • Information gaps in the matrix are flagged as research priorities Alignment • Would fit in the existing tools for performing joint annual reviews within the Ministry of Health, e.g. the Social Economic Status, and cultural context already existing in the multi-sectoral initiatives, public health, institutional capacity, equity • The emphasis on equity is key, since it is an issue in the health sector in Zambia • Creates demand for better systems at different levels
L4D	Step 1: Stakeholder identification and assembling of background information needed for the consultation Step 2: Consultation workshops: These identify priority research issues (as determined by decision-makers) in both the short and longer term; this could also involve discussing the availability or lack of studies on the issues identified Step 3: Translation and sorting; identifying health research priorities and priorities that require synthesis of evidence Step 4: Validate the identified priority issues against similar exercises Step 5: Translate priority issues into research themes; research experts translate the identified research issues into research questions that should and can be answered in order to address the priority issue Step 6: Validate priority research themes with stakeholders; this step ensures that the above translation into research areas correlates to the desires/opinions of stakeholders involved in the consultation of Step 3 – ensuring that the priorities “*truly reflect their expressed views*”	• A strong qualitative/interpretive framework designed to gather and listen above all, not slowing the process down with, for instance, criteria application details • Participatory and transparent • Allows for using combined qualitative and quantitative research techniques • In the Canadian case, the priorities that emerged have received considerable acceptance and funding	Participatory • Involvement of decision-makers is a key component • Promotes clarity in decision-making for policy-makers • Process involves people with knowledge (TWGs) and people with power (decision-makers) • Validation at beginning and end of process • Emphasis on KT – making priorities accessible to different groups within the population The process • The focus on research themes rather than narrower questions allows for flexibility in PS • Situation analysis is important • Two-step validation process, – at the beginning of the process and at conclusion – streamlines M&E processes • KT is important, it makes the process accessible for different populations • Produces both short- and long-term plans • Focus on the future • Can be integrated into existing infrastructure

(Sources: [2–10, 19–29, 40])

*CAM* Combined Approach Matrix, *CHNR* Child Health and Nutrition Research, *ENHR* Essential National Health Research, *JLA* James Lind Alliance, *KT* knowledge translation, *L4D* Listening for Direction, *LIC* low-income country, *M&E* monitoring and evaluation, *PS* priority-setting, *PSP* priority-setting partnership, *RPS* research priority score, *TWG* Technical Working Group

According to the workshop participants, clear, simple, participatory HRPS approaches that involve vulnerable populations and facilitate participants’ understanding by providing them with evidence on which to base their decision-making would be the easiest to adopt within a LIC context. Participants reasoned that the people charged with setting priorities, within this context, often lack the necessary capacity and time to understand complex PS approaches. Therefore, the adoption of clear and simple PS approaches, with step-by-step instructions and clear criteria would reduce the need for experts, promoting local ownership.

Participants also strongly supported participatory PS approaches (a common theme in all the original five PS approaches). There was, however, more support for the method that fostered participation of vulnerable populations who would not normally participate in prioritisation. Hence, the method used in the JLA framework, which focuses on involving patient groups (who were perceived to be vulnerable) in the PS process was greatly desired, more so since they provide the participants with information for decision-making [20]. Most of the above strengths were consistent with those identified in the literature [21–29].

Another strength which was identified by participants as well as the literature was the ease with which a HRPS approach could be aligned with the existing national health system planning processes and infrastructure [5]; this would increase the potential for the process to be sustainable and institutionalised. In this regard, the CAM approach, which recommends multi-sectoral types of evidence at different levels of decision-making (e.g. community, regional, national) was deemed valuable and in line with the national development planning process in Zambia. Furthermore, approaches such as L4D, which facilitate decentralised decision-making by considering priorities from sub-national levels, were perceived favourably since they are aligned with already existing processes and infrastructure and would be easier to adopt and sustain.

### Limitations of the five PS approaches

Table 3 summarises the limitations of the five approaches as identified in the literature review and by workshop participants. There were also similarities between the limitations identified in the literature and the limitations perceived by workshop participants. However, during the workshops, most of the discussion dwelt on the limitations related to the practical applicability (feasibility) and sustainability of using the HRPS approach within LICs such as Zambia.

**Table 3:** Summary of the limitations of the commonly used priority-setting approaches

PS approach	Limitations	
	As identified in the literature	As identified at the workshop, in addition to those in the literature
ENHR	• Process overly based on participant experience, knowledge and views • Identified interventions and research questions are not systematically compiled • Does not clarify which stakeholders to involve and how they can be involved	Might involve several costs: • Hiring facilitation experts, e.g. reviewers reviewing the proposals, and those with needed technical skills to translate research issues into research questions • Dissemination costs • Evaluation costs • Implementation costs (while a PS process is not responsible for implementation costs, they must be considered during the PS process) • Oversight or monitoring is necessary, along with a consideration of relevant costs and hence might not be easily institutionalised within the Ministry
CHNRI	• High risk of bias: the options that are included in the ranking are generated by a small group of experts who may be influenced by their own knowledge and expertise [1, 6] • Does not consider existing government priorities [11] • In most of the cases, the PS process itself was not evaluated [6, 14] • The process is long and complex, which could directly affect response rates [12]	• Complex methodology • Difficult to obtain the right mix of stakeholders depending on the area to be explored • Challenges inherent in getting people to understand how to participate in a reference group • Cost of the PS process: • Meeting costs – bringing stakeholders together • Program Budgeting Marginal Analysis costs – expert might be required • Costs associated with call • Implementation costs
JLA	• The potential inability of patients to respond to surveys and thus registering their perceived treatment uncertainties • Patients may not be equal participants in prioritisation workshops [25] • In some cases, the scope/boundaries of the treatment uncertainty is ill defined and wide ranging [25, 28] • Focus on patients and on disease-specific areas; unclear if the approach is applicable in a broader context • No clear guidance seems to be provided with regards to ranking the treatment uncertainties	• Very difficult to use virtual means to involve necessary populations • Overly biased to treatment needs (and not, for instance, to system needs) • Assumption that the representatives are able to ‘truly represent’ those they claim to represent • How to scale this PS process up to a higher level, e.g. meso- or macro-levels? Costing of PS process: • Human resource costs – consultant if necessary to facilitate process; expert to design survey to collect data on uncertainties • Costs of convening stakeholders meetings • Dissemination costs
CAM	• Lack of information for decision-making in most LICs presents a challenge • It is a difficult method; may be impossible to adequately summarise the wealth of evidence on some topics to a few sentences • Lacks in rigour: the identified priorities are not systematically compiled • Final decision-making performed by a panel of experts who may not be representative • The information needs may necessitate a lot of resources: time and money	• Difficulty in obtaining required evidence Long-term use (especially if the approach will be used again) requires routine, functional systems that collect data (e.g. morbidity, mortality causes) over time • Might require experts on the framework, oversight and facilitation • Complex and multifaceted processes • Diverse skill sets required (e.g. epidemiology, health systems, policy-making) Implementation costs • Costs of validation • Hardware and software costs • Paying highly specialised people to spend time to sit together to figure out the individual components is a time-intensive process
L4D	• Does not provide enough detail on technical issues related to PS process • Requires evidence which may be lacking in some contexts (e.g. the MENA case) • Data collection/analysis did not distinguish between responses given by policy-makers, researchers and representatives of the non-state sector • Purposeful selection of respondents might introduce bias • The lack of criteria creates a question as to how priority issues are identified • Having ‘research experts’ apply seven criteria could introduce bias	• Time consuming process – time is an important commodity • Requires expertise in identification of stakeholders • Costs of facilitator for group process • Time discounting – if you are developing priorities for 10 years the process could be seen as cost effective • Validation of research themes with stakeholders • Investing in pilot projects

(Sources: [2–10, 19–29, 40])

*CAM* Combined Approach Matrix, *CHNR* Child Health and Nutrition Research, *ENHR* Essential National Health Research, *JLA *James Lind Alliance,* L4D *Listening for Direction, *LIC* low-income country, *PS* priority-setting

According to workshop participants, from a practical perspective, the main limitation of the approaches was the potential difficultly in integrating them into the current health system’s planning cycle and infrastructure.

Workshop participants also discussed how approaches that tend to be resource intensive would not be sustainable, given the resource constraints in LICs. For example, while the collection of evidence from different sectors and at different levels (recommended by CAM) was perceived as a strength, the resources that are required to collect all this information may be prohibitive, especially in LICs that lack an established information system [6, 20]. This would limit the feasibility of the HRPS approach and the potential for it to be sustained and institutionalised.

Furthermore, in view of the decentralisation of the health sector, participants thought it was important that HRPS approaches should reflect this by also facilitating decentralised decision-making at sub-national levels. Therefore, approaches that did not provide case studies of sub-national level application were perceived less favourably by the participants.

Although desired, workshop participants observed that most of the HRPS approaches (with the exception of ENHR) lacked a systematic strategy for (1) evaluating the PS process (immediately after setting priorities to gauge if stakeholders were satisfied with the process); (2) monitoring and evaluating the implementation of the set priorities and; (3) enforcing the actual allocation of funds to the identified research priorities. These are key aspects of HRPS, especially in contexts where resources are limited; it is counterintuitive to fail to evaluate the impact and outcomes of HRPS when valuable resources are committed.

### The HRPS approach proposed and validated by the participants

Based on our discussion of the strengths and limitations of the various HRPS approaches, we present a synthesis of the attributes and processes that were deemed desirable and could guide HRPS in Zambia (and potentially other similar LICs) (Table 4). This synthesis is presented in three phases, namely (1) the preparatory phase, including all the activities that must occur before the HRPS process begins; (2) identification of the priorities, namely how a group of stakeholders will arrive at a list of priorities, and the exact type of priorities to be determined (e.g. research questions, research themes, topics, options, or issues etc.); and (3) after PS, namely monitoring, evaluation, funding, enforcement, etc.

**Table 4:** A synthesis of the desired features and process

Pre-requisites for contextualisation, sustainability and institutionalisation • PS process must be owned and led by the country itself; should eventually be integrated into the National Health Strategic plan • Governments should appoint formal health research PS institutions such as NHRA – capable of leading PS processes • Aim to involve technical and non-technical but critical stakeholders at all levels of decision-making and involving multi-sectoral consultations • Include capacity-building for the PS institutions and public sensitisation	
Phase	Process and activities
Preparation	• Determine ownership and leadership; preferably this should be country led and owned • The NHRAs should work with a TWG to lead the process (with clear rationale for the selection), perform a systematic review/literature scan; the TWG should be temporary, depending on the issue under investigation • Decide on level of PS and participants; National PS should cater for the decentralised system, so PS if possible, should start from the bottom-up, i.e. from the district/province to the national level • Identify sources and types of evidence/information/data required Perform a situation analysis • Environment scan (social, economic, political, cultural and global context), decision-maker receptivity and capacity to use the priorities set as a result of the PS process • Determine available resources, e.g. human, financial, availability of experts, in order to inform the choice of method; is there existing capacity to do PS? • Health systems analysis: what other activities in the health system may influence PS? Do these activities support or facilitate the PS process? Do they present specific challenges or hindrances? • Enlist public values either empirically or as identified in the national health research plan (if available) for PS • Conduct a stakeholder analysis and rationale for selection of participants (broad representation of researchers, funders, decision-makers, public) in the PS process • Determine the stakeholder engagement methods (preferably mixed methods tailored to the different stakeholders) • Have an appeals mechanism to ensure procedural fairness • Have a communication strategy to disseminate information • Have an evaluation plan (to evaluate the PS process and the implementation of the priorities • Prior to the face-to-face meeting, send the PS materials (necessary evidence and the details of the PS approaches^a^) to the stakeholders involved in the PS process
Actual PS (preferably face-to-face)	• Present and discuss the evidence collected in the situation analysis • Present and discuss the current approaches used in health research PS • Collectively select the approach and process to guide the PS based on the presented information Considerations for determining the PS methods; the selected PS approach should: • Align with existing Ministry of Health processes and infrastructure • Have potential for institutionalisation and sustainability • Have potential for integration, e.g. with the national strategic plan for health • Facilitate capacity-building (local capacity for setting/leading processes) • Be feasible, with attention to costs (financial, time, human resource skills requirements) • Be adaptable, flexible and easily applied to different issues and levels ▪ Have the ability to produce short- and long-term goals ▪ Be responsive to emerging needs, and be time sensitive ▪ Be participatory (all stakeholders should have an input and views should be respected) • Have explicit criteria (and weighting) for ranking of the research options; already existing criteria should be validated within the local contexts • Define process for actual deciding/ranking of the priorities (if face-to-face, the Nominal Group Technique would be most favourable) Create outputs • This could be a long list of both long- and short-term research priorities • Identify a shorter list, for example ‘the Top 10’
After PS	• Conduct participatory evaluation of the PS process with the stakeholders directly involved in the process, immediately after the PS exercise; use results for improving the next PS cycle • Validate the identified priorities with the relevant stakeholders • Publicise the PS process, the criteria and the validated priorities using appropriate communication mechanisms for different audiences (include international stakeholders) • Implement the priorities: secure and allocate funding for the top 10 research priorities • Under the leadership of the NHRA, annually monitor the implementation of the identified priorities; this should involve comparing funded research within the countries to the identified list of priorities • Evaluate the impact of PS, preferably using a standardised framework; the evaluation should also aim to facilitate institutional capacity strengthening for health research PS

^a^ Kapiriri L et al.’s reference manual synthesizing the literature and demonstrating the potential use would be appropriate [41]

*NHRA* National Health Research Authorities, *PS* priority-setting, TWG Technical **W**orking **G**roups

## Discussion

Contrary to approaches that have already been captured in the literature, this paper presents a process that predominantly included stakeholders who are involved in HRPS in Zambia. Therefore, the discussions and findings focused on contextualising the attributes with the purpose of identifying those that would be sustainable and easily institutionalised within the Zambian context as well as, potentially, in other similar LIC contexts. This paper presents findings from a participatory capacity-building process to identify an acceptable approach to HRPS in Zambia (and other similar LICs). While many of the proposed activities are consistent with those identified in the literature [30, 31], our systematic approach deliberately included the ‘voices’ of the potential users of the approach in the process of its development. These nuances are discussed below.

For example, in the preparation phase, participants emphasised the importance of identifying the ‘right’ level of PS. This is especially relevant in contexts with decentralised systems, as is the case in most LICs. To date, most of the HRPS has been focused at the national level [5–9, 32–35]; however, capacity-building and equitable participation would require that stakeholders from the regions and districts be involved in this process, as was proposed by the workshop participants. This is especially important given that a great deal of health research is conducted within their jurisdictions. The other underlying feature of this process is the emphasis on capacity-building that would involve empowering the institution to ‘own’ the PS process. While COHRED has worked to strengthen NHROs, several of the PS processes for health research that have taken place in sub-Saharan Africa did not demonstrate clear linkages with the NHROs [36, 37]. This not only undermines the credibility of these organisations, it presents a missed opportunity for capacity-building. There is a strong need to support these organisations by ensuring that they take the lead in all PS exercises within their countries. In order to succeed, PS should be part of a broad research management strategy, which should be championed by the NHROS with support from the health and other related ministries.

In the implementation phase, there is no recommendation for a specific PS approach. This is a deliberate omission on our part because we think different approaches would work better for different PS situations. For example, the JLA approach could be the best option for determining treatment-related priorities [38], while CHNRI may prove superior in determining national-level priorities within various research domains, from research priorities within health systems to priorities relating to specific diseases [39]. However, since the current practice has been that the PS facilitators determine and recommend an approach, we propose that, in order to facilitate capacity-building and empower the NHROs, the decision on the framework/approach should be made collectively after all the current approaches have been presented and discussed by the stakeholders, as we demonstrated in the pilot workshops. However, we highlight the need to facilitate an understanding of key considerations when selecting an approach such as feasibility and the potential for integration into current health planning infrastructure during the capacity-building process. We also highlight the need to contextualise the criteria for ranking priorities in order to reflect local values. The HRPS approaches should be flexible so that they can accommodate any locally prioritised values, which may not be included in the standard approaches.

In line with some of the current literature, the proposed approach also emphasises the need for monitoring and evaluation [31]. However, since we also describe the need to build capacity for PS, we discuss the importance of participatory evaluation of the PS process. Preferably, evaluation should be integrated throughout the process and be conducted with the stakeholders involved in PS immediately after the process, as well as a year or two later. An evaluation of the PS process ensures that there is continuous learning about and improvement of the PS process, which will improve and sustain commitment, stakeholder participation and the ongoing institutionalisation of the process. Furthermore, the PS process should be evaluated since it is a waste of scarce resources to invest resources in PS but not to invest in ensuring that the set priorities are actually implemented [18]. Efforts should be devoted to ensuring that there are resources available to fund at least the top ten priorities. There should be active advocacy by the NHRO to ensure that research funders support the research priorities identified through the national PS process. Involvement of research funders in the PS process – from the outset – would greatly facilitate their support.

Evaluation necessitates a standardised form of reporting and explicit parameters. The modified version of the evaluation framework proposed by Kapiriri and Martin [18] could assist with this standardisation. This framework, although developed for evaluating PS for health interventions, has been modified and validated by LIC researchers, and could serve as a resource for stakeholders who want to comprehensively evaluate their HRPS.

### Limitations

Almost all participants were from Zambia, which could limit the applicability of the proposed approach elsewhere, although the similarity of some of the countries in the region may make it applicable.

## Conclusions

This pilot study, conducted in Zambia, provides a summary of the desired attributes for a PS process for health research in LICs. While some of the identified attributes of successful HRPS are consistent with the PS literature, we identified some unique attributes for further discussion. These include a focus on capacity-building by making sure that every PS process starts with introducing the research managers and stakeholders directly involved in the prioritisation process to all existing frameworks, thus ensuring understanding of all HRPS possibilities before choosing the approach to be used. Such an approach not only ensures capacity-building, but cultivates a sense of ownership of the process in LIC stakeholders. Furthermore, the emphasis on seeking a process that is well aligned with existing infrastructure is critical as it ensures sustainability and contributes to the institutionalisation of the PS process.

To date, most of the literature on PS for health research in LICs describes predominantly top-down, one-off, externally-driven exercises. Processes such as the one proposed in this paper, which facilitate local capacity-building, may contribute to ensuring sustainability and institutionalisation of the PS process. There is a need for case studies where such participatory capacity-building approaches are implemented and evaluated.

## Additional files

Additional file 1: Detailed references for the reviewed literature. (DOCX 54 kb)

## Footnotes

1 We defined a ‘framework’ as an approach or series of steps that could take the managers of a PS process from idea (e.g. a desire to identify priority research areas or topics) systematically to the creation of a ranked list of priorities. We excluded ranking strategies such as the Delphi and Nominal group techniques.
