# Supplementary material for: The quest for a framework for sustainable and institutionalised priority-setting for health research in a low-resource setting: the case of Zambia
Source: Health Res Policy Syst. 2018 Feb 17;16:11. doi: 10.1186/s12961-017-0268-7 (PMC5816391; doi:10.1186/s12961-017-0268-7)
Supplement: Supplementary file 1 — Detailed references for the reviewed literature. (DOCX 54 kb) [file 12961_2017_268_MOESM1_ESM.docx]

# **Additional File 1: Detailed references for the reviewed literature**

|  | **PS References for 1997-2014 by Category** |
| --- | --- |
|  | **Category One: concepts, theory, steps of PS Frameworks n=23** |
| **1** | Canadian Health Services Research Foundation. Listening for Direction II: A national consultation on health services and policy issues. CHSRF: Ottawa, 2003. |
| **2** | COHRED. *Priority Setting for Health Research: Toward a management process for low and middle income countries*. COHRED: Geneva. 2006. |
| **3** | COHRED. Essential National Health Research and Priority Setting: Lessons Learned. COHRED: Geneva, 1997. |
| **4** | Cowan K. **The James Lind Alliance—Tackling Treatment Uncertainties Together**. *Journal of Ambulatory Care Management*. 33;3. 241–248. 2010. |
| **5** | Cowan K and Oliver S. *The JLA Guidebook*. 2013. |
| **6** | Dault M, Lomas J and Barer M (2004). Listening for Direction II: National consultation on health services and policy issues for 2004-2007. CHSRF: Ottawa. |
| **7** | de Haan S and Montorzi G. *Priority setting for health research: toward a management process for low– and middle-income countries*. COHRED: Geneva, 2005. |
| **8** | Ghaffar A et al. *The 3D Combined Approach Matrix: An Improved Tool for Setting Priorities in Research for Health.* Global Forum for Health Research: Geneva, 2010. |
| **9** | Ghaffar A. Setting research priorities by applying the combined approach matrix. *Indian Journal of Medical Research*. 2009; 129 |
| **10** | Ghaffar A, de Francisco A, Matlin S, Eds. *The combined approach matrix: a priority setting tool for health research*. Global Forum for Health Research: Geneva, 2004. |
| **11** | Lomas J et al. On being a good listener: setting priorities for applied health services research. 81:3. *The Milbank Quarterly*. 2003. |
| **12** | Montorzi G, de Haan S and IJsselmuiden C. *Priority setting for research for health: a management process for countries*. COHRED: Geneva. 2010. |
| **13** | Okello D et al. A Manual for Research Priority Setting using the ENHR Strategy. The Council on Health Research for Development: Geneva. 2000. |
| **14** | Petit-Zeman S et al. **The James Lind Alliance: tackling research mismatches.***The Lancet* 201:376;667-669. 2010. |
| **15** | Rudan I. Global health research priorities: Mobilizing the developing world. *Public Health*. 126. 2012 |
| **16** | Rudan I et al. Evidence-based priority setting for health care and research: tools to support policy in maternal, neonatal and child health in Africa. *PLoS Medicine*. 7:7. 2010. |
| **17** | Rudan I. The complex challenge of setting priorities in health research investments. *Indian Journal of Medical Research*. 129. 2009 |
| **18** | Rudan I et al. Setting priorities in global child health research investments: universal challenges and conceptual framework. *Croatian Medical Journal*. 49. 2008 |
| **19** | Rudan I et al. Setting priorities in global child health research investments: Guidelines for implementation of the CHNRI method. *Croatian Medical Journal*. 2008; 49: 720-33. |
| **20** | Rudan I et al. Setting priorities in global child health research investments: assessment of principles and practice. *Croatian Medical Journal.* 48. 2007. |
| **21** | Rudan I et al. Childhood pneumonia and diarrhoea: setting our priorities right. *The Lancet Infectious Diseases*. 7. 2007. |
| **22** | The Working Group on Priority Setting. Priority setting for health research: lessons from developing countries. *Health Policy and Planning*. 15:2. 2000. |
| **23** | World Health Organization. *World report on knowledge for better health: strengthening health systems*. Geneva: WHO; 2004. |
|  | **Category Two: PS Framework Application. n=59** |
| **24** | Ali, M et al. A global research agenda for family planning: results of an exercise for setting research priorities. *Bulletin of the World Health Organization*. 92: 2014 |
| **25** | Arbour M et al. (2012). Choosing the Best Child Assessment Instrument for a Specific Context: A Methodology for Engaging Local Experts Applied in Chile. J Dev Behav Pediatr, 33:666–675 |
| **26** | Bahl R et al. Setting research priorities to reduce global mortality from preterm birth and low birth weight by 2015. *Journal of Global Health*. 2:1. 2012. |
| **27** | Bahl R. Research Priorities to Reduce Global Mortality from Newborn Infections by 2015. *The Pediatric Infectious Disease Journal*. 28:1. 2009. |
| **28** | Brown KH et al. Setting priorities for zinc-related health research to reduce children’s disease burden worldwide: An application of the Child Health and Nutrition Research Initiative’s research priority-setting method. *Public Health Nutr*. 2009; 12: 389-96. |
| **29** | Buckley BS, Grant A, Glazener C. **Case study: A patient-clinician collaboration that identified and prioritized evidence gaps and stimulated research development.** *J Clin Epidemiol***.** 66:5; 483-89. 2011 |
| **30** | Buckley BS et al. Prioritizing research: Patients, carers, and clinicians working together to identify and prioritize important clinical uncertainties in urinary incontinence. *Neurourology and Urodynamics*. 29:5;708-714. 2010. |
| **31** | Buckley BS et al. Reaching a consensus on research priorities in urinary incontinence. Nursing Times. Online opinion piece. 2009. |
| **32** | Catto IG et al. An evaluation of oxygen systems for treatment of childhood pneumonia. *BioMed Central*. 11(Suppl 3). 2011 |
| **33** | Chisholm D et al. Scale up services for mental disorders: a call for action. *The Lancet*. Vol 370: 2007. On behalf of the Lancet Global Mental Health Group. |
| **34** | Choudhuri D et al. An evaluation of emerging vaccines for childhood meningococcal disease. *BMC Public Health*. 11(Suppl 3): S29. 2011. |
| **35** | Davila-Seijo P et al. Prioritization of therapy uncertainties in Dystrophic Epidermolysis Bullosa: where should research direct to? an example of priority setting partnership in very rare disorders. *Orphanet Journal of Rare Diseases*. 2013. 8:61 |
| **36** | Dean S et al. (2013). Setting Research Priorities for Preconception Care in Low- and Middle-Income Countries: Aiming to reduce Maternal and Child Mortality and Morbidity. *PLOS Medicine*, 10 (9): e1001508. |
| **37** | El-Jardali F et al. Eliciting policymakers’ and stakeholders’ opinions to help shape health system research priorities in the Middle East and North Africa region. *Health Policy and Planning*. 25. 2010. |
| **38** | Eleftheriadou, V**. Future research into the treatment of vitiligo: where should our priorities lie? Results of the vitiligo priority setting partnership.***British Journal of Dermatology*. 164: 530–536. 2011. |
| **39** | Elwyn G et al. **Identifying and prioritizing uncertainties: patient and clinician engagement in the identification of research questions.** *Journal of Evaluation and Clinical Practice*. 16:3;627-31. 2010. |
| **40** | Flenady V et al. Stillbirths: the way forward in high-income countries. *The Lancet.* Vol 377. 2011. |
| **41** | Fontaine O et al. Setting research priorities to reduce global mortality from childhood diarrhoea by 2015. *PLoS Medicine*. 2009. |
| **42** | Gadsby R et al. **Setting research priorities for Type1 diabetes.** *Diabetic Medicine*. 29:10; 1321-1326, 2012. |
| **43** | George A et al. Setting Implementation Research Priorities to reduce preterm births and stillbirths at the community level. *PLoS Medicine*. 8:1. 2011. |
| **44** | Gregório G et al. Setting priorities for mental health research in Brazil. *Rev Bras Psiquiatr*. 2012 |
| **45** | Hall D et al. **Identifying and prioritizing unmet research questions for people with tinnitus**: the James Lind Alliance Tinnitus Priority Setting Partnership. *Clin. Invest*. 3:1; 21–28. 2013. |
| **46** | Higginson D et al. An evaluation of respiratory administration of measles vaccine for prevention of acute lower respiratory infections in children. *BMC Public Health*. 11 (Suppl 3): S31. 2011 |
| **47** | Hindin MJ, Christiansen CS, Ferguson J. Setting research priorities for adolescent sexual and reproductive health in low- and middle-income countries. *Bulletin of the World Health Organization*. 91 (1). 2013 |
| **48** | Huda T et al. An evaluation of the emerging vaccines and immunotherapy against staphylococcal pneumonia in children. *BMC Public Health*. 11(Suppl 3): S27. 2011. |
| **49** | Jordans MJD, Tol WA, Komproe IH. Mental health interventions for children in adversity: pilot-testing a research strategy for treatment selection in low-income settings. Social Science & Medicine. 2011. 73. 456-466 |
| **50** | Kosek M et al. Directing diarrhoeal disease research towards disease-burden reduction. Journal of Health and Population Nutrition. 2009. 27:3. |
| **51** | Lawn JE et al. Stillbirths: Where? When? Why? How to make the data count? *The Lancet*. 377: 1448-63. 2011. |
| **52** | Lawn JE et al. Setting research priorities to reduce almost one million deaths from birth asphyxia by 2015. *PLoS Medicine*. 2011 |
| **53** | Lienhardt C et al. What research is needed to stop TB? Introducing the TB Research Movement. *PLoS Medicine*. 8(11): 2011. |
| **54** | Lloyd K, White J, Chalmers L. Schizophrenia: **Patients' research priorities get funded.** *Nature.* 487, 432. 2012. |
| **55** | Lloyd K, White J. **Democratizing clinical research.** *Nature* 474:277-278. 2011. |
| **56** | Lophatananon A et al. T**he James Lind Alliance approach to priority setting for prostate cancer research: an integrative methodology based on patient and clinician participation.** British *Journal of Urology International*. 108:7; 1040–1043. 2011. |
| **57** | Morof DF et al. (2014). Neonatal survival in complex humanitarian emergencies: setting an evidence-based research agenda. *Conflict and Health*, 8:8. |
| **58** | Morof DF et al. (2012). Setting operational research priorities for reproductive health in crisis settings: Using the Child Health and Nutrition Research Initiative Methodology. International Journal of Gynecology & Obstetrics. |
| **59** | Nair H et al. (2013). An evaluation of the emerging vaccines against influenza in children. *BMC Public Health*, 12(Suppl 3): S14. |
| **60** | Nair H et al. An evaluation of the emerging interventions against Respiratory Syncytial Virus (RSV) – associated acute lower respiratory infections in children. *BioMed Central*. 11(Suppl3). 2011. |
| 61 | Owlia, P et al. Health research priority setting in Iran: Introduction to a bottom up approach. *Journal of Research in Medical Sciences*, 16(5): 691-698. 2011. |
| **62** | Parlour R and Slater P. Developing Nursing and Midwifery Research Priorities: A Health Services Executive (HSE) North West Study. *Worldviews on Evidence-Based Nursing*. 11(3). 2014 |
| **63** | Pollock A et al. Development of a new model to engage patients and clinicians in setting research priorities. *Journal of Health Services, Research and Policy*. 19:1. 2014. |
| **64** | Pollock A et al. **Top ten research priorities relating to life after stroke.** The Lancet Neurology. 11:3;209. 2012 |
| **65** | Pollock A et al. **Top 10 research priorities relating to life after stroke - consensus from stroke survivors, caregivers, and health professionals.** *Int J Stroke*. Dec 11. 2012. |
| **66** | Rollins N et al. (2014). Prioritizing the PMTCT Implementation Research Agenda in 3 African Countries: Integrating and Scaling up PMTCT Through Implementation Research (INSPIRE). Journal of Acquired Immunodeficiency Syndrome, 67(2): s108-s113. |
| **67** | Rudan I et al. Setting priorities for development of emerging interventions against childhood pneumonia, meningitis and influenza. *Journal of Global Health*. 2(1). 2012. |
| **68** | Rudan I et al. Setting priorities to reduce global mortality from childhood pneumonia by 2015. *PLoS Medicine*. 8:9, 2011. |
| **69** | Sekar N et al. Research Options for Controlling Zoonotic Disease in India, 2010-2015. *PLoS One*. 6(2). 2011. |
| **70** | Souza JP et al. (2014). Maternal and perinatal health research priorities beyond 2015: an international survey and prioritization exercise. *Reproductive health*, 11:61. |
| **71** | Thomas R et al**. Identifying and prioritizing epilepsy treatment uncertainties.** *J Neural Neurosurg Psychiatry*. 81:918-921. 2010. |
| **72** | Tol WA et al. Research Priorities for Mental Health and Psychosocial Support in Humanitarian Settings. *PLoS Medicine.* 8(9). 2011. |
| **73** | Tomlinson M et al. Research priorities for health of people with disabilities: an expert opinion exercise. *The Lancet*. Vol 374. November 28, 2009. |
| **74** | Tomlinson M et al. Setting priorities for global mental health research. *Bulletin of the World Health Organization*. 87. 2009 |
| **75** | Tomlinson M et al. Setting priorities in child health research investments for South Africa. *PLoS Medicine*. 4:8. 2007. |
| **76** | Tyndale-Biscoe S, Malcolm E, Gnanapragasam VJ. **Setting priorities for prostate cancer research.** *Trends in Urology & Men's Health*. 3:1; 31-33. 2012. |
| **77** | Uneke CJ et al. Research priority setting for health policy and health systems strengthening in Nigeria: the policymakers’ and stakeholders’ perspective and involvement. *Pan-African Medical Journal*. 2013 |
| **78** | Walley J, et al. Primary Health Care: making Alma Ata a reality. *The Lancet*. 2008; 372: 1001-7. 10. |
| **79** | Wazny K et al. (2014). Setting global research priorities for integrated community case management (iCCM): Results from a CHNRI (Child Health and Nutrition Research Initiative) exercise. Journal of Global Health, 4(2). |
| **80** | Wazny K et al. (2013). Setting Research Priorities to Reduce Mortality and Morbidity of Childhood Diarrhoeal Disease in the Next 15 Years. PLOS Medicine, 10 (5): e1001446. |
| **81** | Webster J et al. An evaluation of emerging vaccines for childhood pneumococcal pneumonia. *BMC Public Health*. 11 (Suppl 3): S26. 2011. |
| **82** | Yoshida S et al. (2014). Newborn health research priorities beyond 2015. *The Lancet*, 384:e27-e29. |
|  | **Category Three: Specific PS Aspects. n=26** |
| **83** | Bruni RA et al. Public involvement in the priority setting activities of a wait time management initiative: a qualitative case study. *BMC Health Services Research*. 7:186. 2007. |
| **84** | Campbell S. *Deliberative Priority Setting*. A CIHR Knowledge Translation Module. Canadian Institutes of Health Research: Ottawa, 2010 |
| **85** | Chanda-Kapata P, Campbell S, Zarowsky C. Developing a national health research system: participatory approaches to legislative, institutional, and networking dimensions in Zambia. Health Research Policy and Systems. 6;10:17. 2012. |
| **86** | Chapman, E. et al. A survey study identified global research priorities for decreasing maternal mortality. *Journal of Clinical Epidemiology*. 64: 314-324. 2014 |
| **87** | Daniels N and Sabin JE. Accountability for reasonableness: an update. *BMJ.* 337. 2008. |
| **88** | Daniels N and Sabin JE. *Setting Limits Fairly: Can We Learn to Share Medical Resources?* Oxford University Press: Oxford. 2002. |
| **89** | Daniels N. Accountability for reasonableness: establishing a fair process for priority setting is easier than agreeing on principles. *BMJ*. 321. 2000. |
| **90** | Daniels N and Sabin JE. The ethics of accountability in managed care reform. *Health Affairs*. 17:5. 1998. |
| **91** | Kapiriri L and Martin DK. Successful Priority Setting in LMICs: A Framework for Evaluation. *Health Policy Analysis*. 2009. |
| **92** | Kapiriri L and Martin DK. A Strategy to Improve priority setting in developing countries. *Health Care Analysis*. 15. 2007. |
| **93** | Kapiriri L, Norheim O, Martin DK. Priority setting at the micro-, meso- and macro-levels in Canada, Norway and Uganda. *Health Policy*. 82. 2007 |
| **94** | Kapiriri L et al. Setting priorities in global child health research investments: Addressing the values of the stakeholders. *Croatian Medical Journal*. 2007; 48: 618-27. |
| **95** | Lavis JN et al. SUPPORT Tools for evidence-informed health Policymaking (STP) 3: Setting priorities for supporting evidence-informed policymaking. *Health Research Policy and Systems*. 2009. |
| **96** | Lenaway J, et al. Public health systems research: setting a national agenda. *American Journal of Public Health*. 96:3. 2006. |
| **97** | McGregor S et al. How are health research priorities set in low and middle income countries? A systematic review of published reports. *PLoS One*. 2014 Oct 2;9(9) 2014. |
| **98** | Nuyens Y. Setting priorities for health research: lessons from low- and middle-income countries. *Bulletin of the World Health Organization*. 85:4. 2007 |
| **99** | Ranson MK and Bennett SC. Priority setting and health policy and systems research. *Health Research Policy and Systems*. 7:27. 2009. |
| **100** | Reveiz L et al. Comparison of national health research priority-setting methods and characteristics in Latin America and the Caribbean, 2002-2012. *Rev Panam Salud Publica*. 2013. |
| **101** | Sabik LM, Lie RK. Priority setting in health care: Lessons from the experiences of eight countries. *International Journal for Equity in Health*. 7:4. 2008. |
| **102** | Schmidt W-P. Setting Priorities in Diarrhoeal Disease Research: Merits and Pitfalls of Expert Opinion. *Editorial* in Journal of Health and Population Nutrition. 2009. 27:3 |
| **103** | Sibbald SL et al. Priority setting: what constitutes success? A conceptual framework for successful priority setting. *BMC Health Services Research.* 9:43. 2009. |
| **104** | Smith N et al. Identifying research priorities for health care priority setting: a collaborative effort between managers and researchers. *BMC Health Services Research*. 9:165. 2009. |
| **105** | Stewart R et al. Patients’ and Clinicians’ Research Priorities. *Health Expectations*. 14:4; 439-448. 2011 |
| **106** | Tomlinson M et al. A review of selected research priority setting processes at national level in low– and middle-income countries: towards fair and legitimate priority setting. *Health Research Policy and Systems*. 9:19. 2011. |
| **107** | Viergever RF et al. A checklist for health research priority setting: nine common themes of good practice. *Health Research Policy and Systems*. 8:36. 2010 |
| **108** | 1. Yoshida S. Approaches, tools and methods used for setting priorities for health research in the 21^st^ century. Journal of Global Health. 6:1. 2016. |

**Limitations**

The principle limitation of this stocktaking report lies in not reviewing or considering the grey literature. We are integrating that literature in other aspects of our research project (e.g. within the manual we are developing to guide PS processes in LICs).

# **Appendix One**

**Table 1: Literature Search in Four Databases**

| **Literature Search: PS for Health Research 1997–2014** | | | |
| --- | --- | --- | --- |
| **1. Google Scholar -** <https://scholar.google.com/> | | | |
| **Search Terms** | **Results: # of papers 1997–2014** | **# of Abstracts selected for review** | **# of Papers actually reviewed** |
| “priority setting for health research” | 160 | 81 | 62 |
| “priority setting for health research” in "developing countries" | 154 | 94 | 48 |
| “priority setting for health research” in “low-income countries” | 149 | 94 | 47 |
| **2. PubMed -** <http://www.ncbi.nlm.nih.gov/pubmed/advanced> | | | |
| “priority setting for health research” | 1508 | 102 | 87 |
| “priority setting for health research” in "developing countries" | 96 | 67 | 52 |
| “priority setting for health research” in “low-income countries” | 48 | 38 | 28 |
| **3. Ovid – http://ovidsp.tx.ovid.com.libaccess.lib.mcmaster.ca/** | | | |
| “priority setting for health research” | 14 | 13 | 12 |
| “priority setting for health research” in "developing countries" | 0 | 0 |  |
| “priority setting for health research” in “low-income countries” | 0 | 0 |  |
| **Total Papers Reviewed** |  |  | **108** |

Note that in “the number of papers actually reviewed” column, there are duplicates (i.e. the same paper identified in more than one database, and/or by more than one search string).
